# Ultrafast control over chiral sum-frequency generation

**DOI:** 10.1126/sciadv.adj1429

**Published:** 2023-08-18

**Authors:** Joshua Vogwell, Laura Rego, Olga Smirnova, David Ayuso

**Affiliations:** ^1^ Department of Physics, Imperial College London, SW7 2AZ London, UK.; ^2^ Universidad de Salamanca, 37008 Salamanca, Spain.; ^3^ Departamento de Química, Universidad Autónoma de Madrid, 28049 Madrid, Spain.; ^4^ Max-Born-Institut, Max-Born-Str. 2A, 12489 Berlin, Germany.; ^5^ Technische Universität Berlin, 10623 Berlin, Germany.

## Abstract

We introduce an ultrafast all-optical approach for efficient chiral recognition that relies on the interference between two low-order nonlinear processes that are ubiquitous in nonlinear optics: sum-frequency generation and third-harmonic generation. In contrast to traditional sum-frequency generation, our approach encodes the medium’s handedness in the intensity of the emitted harmonic signal, rather than in its phase, and it enables full control over the enantiosensitive response. We show how, by sculpting the sub-optical-cycle oscillations of the driving laser field, we can force one molecular enantiomer to emit bright light while its mirror twin remains dark, thus reaching the ultimate efficiency limit of chiral sensitivity via low-order nonlinear light-matter interactions. Our work paves the way for ultrafast and highly efficient imaging and control of the chiral electronic clouds of chiral molecules using lasers with moderate intensities, in all states of matter: from gases to liquids to solids, with molecular specificity and on ultrafast time scales.

## Teaser

An all-optical approach for distinguishing left- and right-handed chiral molecules on ultrafast timescales is introduced.

## INTRODUCTION

Chirality is a universal type of asymmetry that naturally arises in molecules, optical fields, viruses, or even galaxies. Similar to a chiral glove that would either fit our left or right hand, but not both, the two nonsuperimposable mirror–reflected versions of a chiral molecule (enantiomers) can behave very differently when they interact with another chiral entity, e.g., another chiral molecule. Since most molecules supporting biological life are chiral, methods for detecting, quantifying, and manipulating molecular chirality are of great importance and interest, particularly in biochemical and pharmaceutical contexts.

Traditional chiroptical methods (*1*–*4*) rely on weak linear effects that arise beyond the electric-dipole approximation, posing substantial limitations for detecting dilute samples and for ultrafast spectroscopy (*5*). This challenge (*6*) can be addressed by developing and applying approaches that rely exclusively on the molecular response to the local polarization of the electric field vector of an electromagnetic wave (*6*–*49*). One strategy is to record the forward-backward asymmetry in the photoelectron angular distributions upon ionization with circularly polarized light (*8*–*21*). This method, originally proposed (*8*) and implemented (*9*–*13*) in the linear regime, has been extended to the nonlinear (*13*–*25*) (multiphoton) regime, where the use of two-color light pulses enables unique opportunities for coherent control (*22*–*25*), and recently observed in anions (*50*).

The nonlinear regime of purely electric-dipole light-matter interactions also enables efficient all-optical chiral spectroscopy (*33*–*49*). The same principle can be applied to microwave fields (*28*–*32*), as predicted by Kral and coworkers (*51*, *52*), and reformulated for molecular rotations by several groups (*53*, *54*).

Sum-frequency generation (SFG) (*33*–*41*) is a well-established nonlinear spectroscopic method driven by purely electric-dipole interactions. It uses two incident laser beams with frequencies ω_1_ ≠ ω_2_ propagating in different directions, with wave vectors ${\hat{k}}_{1}$ and ${\hat{k}}_{2}$ and polarizations ${\hat{e}}_{1}$ and ${\hat{e}}_{2}$, to drive a second-order response in the medium at frequency ω_3_ = ω_1_ + ω_2_. SFG has been successfully applied to characterise the structure and dynamics of biologically relevant systems at interfaces (*55*), from achiral organic molecules (*56*) and chiral amino acids (*57*) to larger biological structures (*55*) including peptides and proteins (*58*–*65*) and DNA (*66*–*68*).

SFG is strictly forbidden in the bulk of isotropic media, such as randomly oriented molecules, unless they are chiral. Therefore, SFG from molecules in the gas or liquid phase provides a unique and background-free signature of molecular chirality. The induced polarization associated with SFG in isotropic media is orthogonal to the polarization plane of the total electric field vector of the driving field: ${\hat{e}}_{3}={\hat{e}}_{1}\times {\hat{e}}_{2}$, and it leads to emission of light at frequency ω_3_ in the direction of ${\hat{k}}_{3}={\hat{k}}_{1}+{\hat{k}}_{2}$. Since it is driven by purely electric-dipole interactions, the induced signals can be strong if ω_3_ is close to resonance (*35*). However, the intensity of SFG is not enantiosensitive—the molecular handedness remains hidden in the phase of the emitted radiation.

To measure the phase of SFG and, thus, the medium’s handedness, one can make it interfere with a reference signal using a local oscillator (*41*). The achiral reference can also be generated from the chiral sample itself, making the near-field intensity enantiosensitive. To this end, one can take advantage of magnetic interactions (*34*) or use a constant electric field (*37*), although these strategies offer limited enantiosensitivity and opportunities for control.

Here, we introduce an all-optical approach for efficient chiral discrimination that relies on the interference between two low-order nonlinear processes: chiral SFG and achiral third-harmonic generation (THG). We show how, by sculpting the subcycle oscillations of the laser’s electric field vector, we can control the ultrafast optical response of the molecules in a highly enantiosensitive manner: quenching the low-order nonlinear response of one molecular enantiomer while maximizing it in its mirror twin. This work shows that high enantiosensitivity is not limited to highly nonlinear processes (*42*–*48*): It can also be achieved in the perturbative regime.

## RESULTS AND DISCUSSION

Chiral SFG can be efficiently driven by any combination of frequencies ω_1_ ≠ ω_2_, as long as ω_3_ = ω_1_ + ω_2_ is close to resonance (*35*). Let us impose ω_2_ = 2ω_1_ and, thus, ω_3_ = 3ω, with ω = ω_1_ being the fundamental frequency, and consider the next-order nonlinear process: THG. The medium can efficiently absorb three photons of frequency ω from the first beam, still at relatively low laser intensities, which leads to achiral polarization at frequency 3ω (by achiral, we mean that the induced polarization has identical amplitude and phase in opposite molecular enantiomers). Can this achiral response of the molecule interfere with the sum-frequency response to produce an enantiosensitive interference? Unfortunately, momentum conservation dictates that, while the SFG and THG signals have the same frequency, they are emitted in different directions. The THG signal copropagates with the ω beam, as $k_{\mathrm{THG}}=3k_{\omega }$, whereas the SFG signal is emitted in between the two driving beams because **k**_SFG_ = **k**_ω_ + **k**_2ω_. In the following, we show how a relatively simple modification of the original SFG setup allows us to overcome this limitation.

**Fig. 1.**
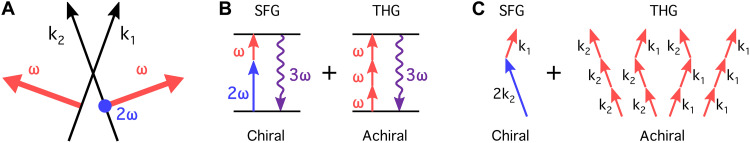
Enantiosensitive SFG. (**A**) The proposed optical setup combines a linearly polarized beam with frequency ω and a second beam that carries cross-polarized ω and 2ω frequencies. The ω frequency components are polarized in the plane of propagation, and the 2ω component is polarized orthogonal to this plane. (**B**) Multiphoton pathways describing chiral SFG (left) and achiral THG (right). The induced polarization associated with SFG has the same amplitude and opposite phase in opposite molecular enantiomers, $P_{\mathrm{SFG}}^{L}=-P_{\mathrm{SFG}}^{R}$, whereas the polarization associated with THG is identical, $P_{\mathrm{THG}}^{L}=P_{\mathrm{THG}}^{R}$. (**C**) Multiphoton diagrams describing momentum conservation in SFG (left) and THG (right).

The proposed optical setup combines a linearly polarized beam with frequency ω and a second beam that carries cross-polarized ω and 2ω frequencies. The ω components are polarized in the plane of propagation, whereas the 2ω component is polarized orthogonal to this plane (see Fig. 1A). The laser field can be written as

$$E=ℜ\{E^{\left(1\right)}+E^{\left(2\right)}\}$$ (1)

$$E^{\left(1\right)}=E_{\omega }^{\left(1\right)}e^{-i\omega t+ik_{1}\cdot r}{\hat{e}}_{1}$$ (2)

$$E^{\left(2\right)}=E_{\omega }^{\left(2\right)}e^{-i\omega t+ik_{2}\cdot r}{\hat{e}}_{2}+E_{2\omega }^{\left(2\right)}e^{-2i\omega t-i\phi_{2\omega }+2ik_{2}\cdot r}\hat{z}$$ (3)

where $E_{\omega }^{\left(1\right)}$, $E_{\omega }^{\left(2\right)}$, and $E_{2\omega }^{\left(2\right)}$ are the electric field amplitudes including the temporal and spatial Gaussian envelopes and ϕ_2ω_ is the two-color phase delay in the second beam.

Let us emphasize the key differences between our setup (Fig. 1A) and traditional SFG configurations. First, we set the ratio between the two input frequencies to ω_2_/ω_1_ = 2. Second, we add the fundamental ω frequency to the second beam. These two features determine a fundamental difference in the properties of the created field. In traditional SFG setups, the polarization of the electric field vector is confined to a plane and, therefore, achiral within the electric-dipole approximation. In our setup, the combination of the two beams creates a locally chiral field: The polarization of the electric field vector draws a (three-dimensional) chiral trajectory in time (*43*).

The generated locally chiral field can drive a strongly enantiosensitive response in a medium of randomly oriented chiral molecules via interference between SFG and THG (see Fig. 1B). The SFG pathway is as in traditional SFG implementations (see Fig. 1C, left). However, by adding the ω frequency to the second beam, we open new THG pathways (see Fig. 1C, right). The medium can now absorb the three ω photons from the same beam or two photons from one beam and one from the other, giving rise to emission of achiral THG in four different directions. One of these pathways, the one involving absorption of one photon from the first beam and two photons from the second beam, leads to achiral THG emission exactly in the same direction as the chiral SFG signal (Fig. 1C). The two contributions can now interfere, making the intensity of emission strongly enantiosensitive.

**Fig. 2.**
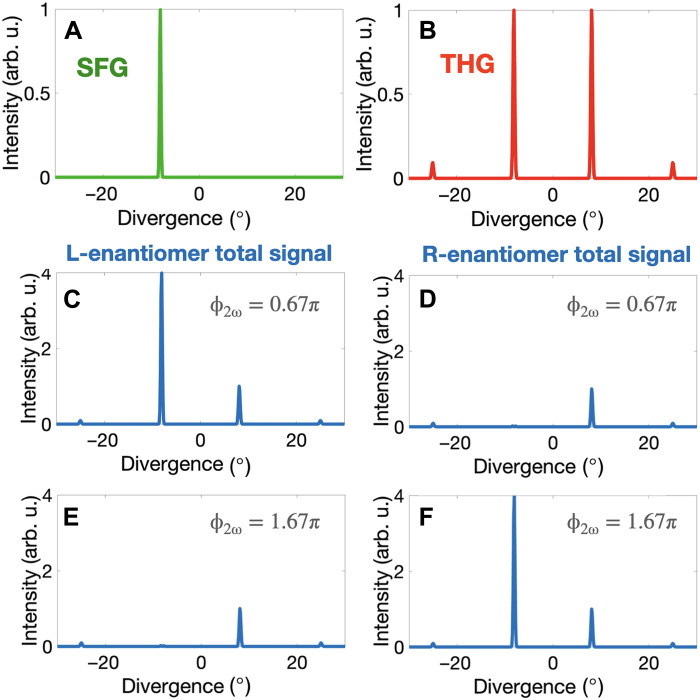
Enantiosensitive SFG in propylene oxide. (**A** and **B**) Intensity at frequency 3ω in the far field associated with chiral SFG (A) and achiral THG (B). (**C** to **F**) Total intensity emitted from L (C) and (E) and R (D) and (F) propylene oxide, resulting from adding the chiral SFG (A) and achiral THG (B) contributions, for ϕ_2ω_ = 0.67π (C) and (D) and ϕ_2ω_ = 1.67π (E) and (F). Laser parameters: $I_{\omega }^{\left(1\right)}=I_{\omega }^{\left(2\right)}=3\times 10^{12}$ W/cm^2^, $I_{2\omega }^{\left(2\right)}=7\times 10^{11}$ W/cm^2^, pulse duration of 7 fs, and opening angle 2α = 50°. arb. u., arbitrary units.

To demonstrate our proposal, we have performed state-of-the-art numerical simulations in randomly oriented propylene oxide molecules (see Methods). We have considered the following laser parameters: intensity of the ω field in the first and second beams $I_{\omega }^{\left(1\right)}=I_{\omega }^{\left(2\right)}=3\times 10^{12}$ W/cm^2^, intensity of the 2ω component $I_{2\omega }^{\left(2\right)}=7\times 10^{11}$ W/cm^2^, pulse duration of 7 fs, and opening angle 2α = 50°. Figure 2 (A and B) shows the intensity of the two contributions to emission at frequency 3ω in the far field (SFG and THG). As already anticipated, the chiral SFG contribution is emitted at a divergence angle −$\arcsin\left(\frac{\sin(\alpha )}{3}\right)=-8.1{}^{\circ }$ (Fig. 1C, left), whereas the achiral THG profile shows four peaks, at angles of −25.0°, −8.1°, 8.1°, and 25.0°, corresponding to the four possible *k*-vectors’ combinations (Fig. 1C, right).

The intensity profiles of the SFG and THG contributions are, individually, not enantiosensitive (Fig. 2, A and B). However, since the SFG contribution is out of phase in opposite molecular enantiomers, the total intensity of emission, resulting from adding the two contributions, becomes strongly enantiosensitive (see Fig. 2, C to F). We have tuned the amplitude of the 2ω component of the driving field so the SFG contribution and the THG contribution at an emission angle of −8.1° have equal amplitude. Then, we adjust the two-color phase delay in the driving field and control the phase of SFG, achieving full control over the enantiosensitive interference and, thus, over the intensity of emission at frequency 3ω. For ϕ_2ω_ = 0.67π, the SFG and THG contributions interfere constructively in the left-handed molecules (Fig. 2C) and destructively in the right-handed molecules (Fig. 2D). Changing the two-color delay by π, i.e., setting ϕ_2ω_ = 1.67π, changes the phase of the SFG contribution by π, leading to the opposite effect: suppression from the left-handed molecules (Fig. 2E) and strong emission from the right-handed molecules (Fig. 2F).

**Fig. 3.**
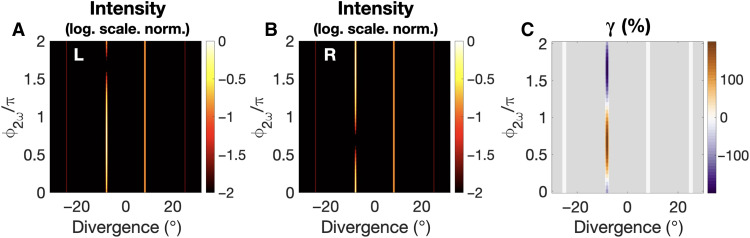
Enantiosensitive control of SFG. (**A** and **B**) Intensity at frequency 3ω emitted from left-handed (A) and right-handed (B) propylene oxide as a function of the divergence angle and the two-color phase delay. (**C**) Dissymmetry factor γ = 2(*I*_L_ − *I*_R_)/(*I*_L_ + *I*_R_). Gray color indicates no intensity of emission. See caption of Fig. 2 for laser parameters.

Figure 3 shows how we can control the intensity of emission in a highly enantiosensitive manner. Because the polarization associated with SFG is out of phase in opposite enantiomers, the values of the two-color delay that maximize and quench emission at an angle of −8.1° from the L (Fig. 3A) and R (Fig. 3B) enantiomers are shifted by π. Note that the specific values of the two-color delay that optimize the enantiosensitive interference depend on the relative phase between the SFG and THG contributions to light-induced polarization, which record the anisotropy of the chiral molecular potential and are molecule-specific quantities. Thus, by adjusting the laser parameters, including the input laser frequencies, intensities, and phase delay, one could drive an equally strong enantiosensitive response in any chiral molecule.

To quantify the degree of enantiosensitivity in the nonlinear optical response, we use a standard definition of the dissymmetry factor, $\gamma =\frac{I_{L}-I_{R}}{(I_{L}+I_{R})/2}$. As shown in Fig. 3C, γ reaches the limits of ±200%. That is, we can maximize emission from the left-handed molecules and fully quench it in the right-handed molecules (γ = 200%), or vice versa (γ = −200%), by adjusting the two-color phase delay in the second beam. Because the two-color delay defines the local handedness of the locally chiral field, it controls the enantiosensitive response of the chiral molecules.

Note that while the intensity of emission at an angle of −8.1° is strongly enantiosensitive and can be fully controlled, the peak at 8.1° is completely independent on both the molecular handedness and the two-color delay. This is due to the different phase matching conditions for SFG and THG, as can be understood in terms of conservation of momentum (Fig. 1), which dictate that the peak at 8.1° is solely due to THG. Therefore, this peak constitutes a constant reference that can be used for calibration purposes and for reducing the noise in experimental measures.

Our proposal constitutes a simple, yet highly efficient, all-optical approach for chiral discrimination that relies on the interference between low-order nonlinear phenomena (SFG and THG) that are ubiquitous in nonlinear optics. They have both been widely recorded in chiral and achiral media, in isotropic and anisotropic samples, and in the gas, liquid, and solid phases of matter. However, despite the general importance of chiral molecules, to the best of our knowledge, these two phenomena had not been combined to achieve efficient chiral discrimination.

Our approach takes advantage of the tremendous capabilities of modern optical technology for sculpting the polarization of light with sub-optical-cycle temporal resolution. By controlling the two-color delay in the proposed optical setup, we can tailor the chirality of the driving field to maximize the nonlinear response of a selected molecular enantiomer while suppressing it in its mirror twin. The possibility of driving strongly enantiosensitive interactions via low-order nonlinear processes creates tremendous opportunities for imaging and controlling molecular chirality on ultrafast time scales using laser fields with gentle intensities, as well as for developing enantiosensitive optical traps and tweezers.

## METHODS

### Single-molecule response of randomly oriented propylene oxide

The ultrafast electronic response of randomly oriented propylene oxide to the proposed driving field was evaluated using real-time time-dependent density functional (TDDFT) theory in Octopus (*69*–*72*). We used the local density approximation (*73*–*75*) to account for electronic exchange and correlation effects, together with the averaged density self-interaction correction (*76*). The 1-s orbitals of the carbon and oxygen atoms were described by pseudopotentials. We expanded the Kohn-Sham orbitals and the electron density onto a spherical basis set of radius *R* = 41.9 atomic units (a.u.), with Δ*x* = 0.4 a.u. of spacing between adjacent grid points, and used a complex absorbing potential of a width of 20 a.u. and a height of −0.2 a.u. to avoid unphysical reflection effects. In our TDDFT simulations, we used a two-color cross-polarized driving field

$$E_{0}(t)=a(t)[E_{\omega }^{\left(0\right)}\cos(\omega t)\hat{x}+E_{2\omega }^{\left(0\right)}\cos(2\omega t)\hat{z}]$$ (4)

where $E_{\omega }^{\left(0\right)}=E_{2\omega }^{\left(0\right)}=0.00534$ a.u. are the electric field amplitudes, ω = 0.126 a.u. is the fundamental frequency, and *a*(*t*) is sine-squared flat-top envelope of eight laser cycles of the fundamental frequency, with 2 cycles to rise up, 4 cycles of constant amplitude, and 2 cycles to go down.

We run TDDFT simulations for 208 different molecular orientations to evaluate the induced polarization in the randomly oriented ensemble

$$P_{0}=\frac{1}{8\pi^{2}}\int_{0}^{2\pi }\int_{0}^{\pi }\int_{0}^{2\pi }P_{\phi \theta \chi }\;\sin(\theta )d\phi \;d\theta \;d\chi$$ (5)

where ϕ, θ, and χ are the Euler angles and **P**_ϕθχ_ is the polarization induced in a particular molecular orientation in the laboratory frame. We used the Lebedev quadrature (*77*) of order 7 to integrate over ϕ and θ, thus using 26 points to sample the two angles, and the trapezoid method to integrate numerically over χ, using 8 angular points.

### Laser field in the interaction region

The laser field was modeled using Eqs. 1 to 3, with $k_{1,2}=\frac{2\pi }{\lambda }[\pm \sin(\alpha )\hat{x}+\cos(\alpha )\hat{y}]$, where λ = 362 nm is the fundamental wavelength. The two beams propagate in the *xy* plane, at angles ±α with respect to the *y* axis. We assume a thin medium, which could be realized using a flat liquid microjet (*78*–*80*), and thus neglect the spatial modulation of the field properties along the propagation direction, and set the position of the thin sample at *y* = 0. Setting intensity of the ω frequency component to be the same in the two beams, the total electric field vector of the laser at *y* = *z* = 0 can be written as

$$E(x,t)=a(t)\{E_{x}(x)\cos(\omega t)\hat{x}+E_{y}(x)\sin(\omega t)\hat{y}+E_{z}(x)\cos[2\omega t+\phi (x)]\hat{z}\}$$ (6)

with

$$E_{x}(x)=2E_{\omega }\cos(\alpha )\cos(k\sin\alpha \;x)e^{-x^{2}/w^{2}}$$ (7)

$$E_{y}(x)=2E_{\omega }\sin(\alpha )\sin(k\sin\alpha \;x)e^{-x^{2}/w^{2}}$$ (8)

$$E_{z}(x)=E_{2\omega }e^{-x^{2}/w^{2}}$$ (9)

$$\phi (x)=\phi_{2\omega }+2k\sin(\alpha )\;x$$ (10)

where *E*_ω_ and *E*_2ω_ are the electric field amplitudes, *w* is the waist of the Gaussian beams, and ϕ_2ω_ is the two-color phase delay in the second beam.

### Nonlinear response in the near field

The enantiosensitive response of the molecules at frequency 3ω results from the interference between two contributions to light-induced polarization: chiral SFG and achiral THG (see Fig. 1). The SFG and THG contributions were obtained by projecting the induced polarization in the frequency domain **P**_0_ over the *x* and *y* axes, $P_{0}^{\mathrm{THG}}=[P_{0}(3\omega )\;\cdot \hat{x}]\hat{x}$ and $P_{0}^{\mathrm{SFG}}=[P_{0}(3\omega )\;\cdot \hat{y}]\hat{y}$.

Note that this procedure neglects the contribution of the *y* component of the electric field vector to both THG and SFG, which considerably reduces the computational cost of the simulations. As a result, $P_{0}^{\mathrm{THG}}$ and $P_{0}^{\mathrm{SFG}}$ are linearly polarized along the *x* and *y* axes, respectively, and not elliptically polarized in the *xy* plane. Including the *y* component of the electric field vector in the simulations would make both **P**^THG^ and **P**^SFG^ elliptically polarized in the *xy* plane, but it would not lead to significant changes in the results presented in Figs. 2 and 3.

To model the nonlinear response of the molecules across the interaction region, we assumed that the induced polarization depends on the field amplitudes according to the number of absorbed photons (see Fig. 1)

$$P^{\mathrm{THG}}(x)=P_{0}^{\mathrm{THG}}{\left(\frac{E_{x}(x)}{E_{\omega }^{\left(0\right)}}\right)}^{3}$$ (11)

$$P^{\mathrm{SFG}}(x)=P_{0}^{\mathrm{SFG}}\frac{E_{x}(x)}{E_{\omega }^{\left(0\right)}}\frac{E_{z}(x)}{E_{2\omega }^{\left(0\right)}}e^{i\phi (x)}$$ (12)

where $E_{\omega }^{\left(0\right)}$ and $E_{2\omega }^{\left(0\right)}$ are the electric field amplitudes used in the TDDFT simulations (Eq. 4), and *E*_*x*_, *E*_*z*_, and ϕ are the field amplitudes and phase delay across the transverse coordinate *x* (Eq. 6). We performed calculations for the right-handed enantiomer of propylene oxide and obtained the results for the left-handed enantiomer using symmetry arguments.

### Enantiosensitive intensity in the far field

The far-field image was evaluated using Fraunhofer diffraction

$$E(\kappa )\propto 9\omega^{2}\int_{-\infty }^{\infty }P_{⊥}(x)\;e^{-iKx}dx$$ (13)

where **P**_⊥_ is obtained by projecting the total polarization at frequency 3ω, **P** = **P**^THG^ + **P**^SFG^ (Eqs. 11 and 12), onto the plane that is orthogonal to the propagation direction $\hat{k}=\cos(\kappa )\hat{x}+\sin(\kappa )\hat{y}$, and $K=\frac{3\omega }{c}\sin(\kappa )$, with κ being the far-field angle (divergence), ω being the fundamental frequency, and *c* being the speed of light in vacuum.
